# Aberrant Frequency of IL-10-Producing B Cells and Its Association with Treg/Th17 in Adult Primary Immune Thrombocytopenia Patients

**DOI:** 10.1155/2014/571302

**Published:** 2014-06-26

**Authors:** Fanli Hua, Lili Ji, Yanxia Zhan, Feng Li, Shanhua Zou, Lihang Chen, Song Gao, Ying Li, Hao Chen, Yunfeng Cheng

**Affiliations:** ^1^Department of Haematology, Zhongshan Hospital, Fudan University, 180 Fenglin Road, Shanghai 200032, China; ^2^Department of Haematology, Jinshan Hospital, Fudan University, Shanghai 201508, China; ^3^Department of Internal Medicine, John A. Burns School of Medicine, University of Hawaii, HI 96813, USA; ^4^Department of Cardiothoracic Surgery, Tongji Hospital, Tongji University, Shanghai 200065, China; ^5^Research Centre, Zhongshan Hospital, Fudan University, Shanghai 200032, China

## Abstract

*Background*. Regulatory B cells (Breg) are a distinct B cell subset with immunoregulatory properties. Pivotal to Breg function is interleukin-10. This study was to investigate the role of IL-10-producing B cell (B10) and its association with Treg and Th17 subsets in immune thrombocytopenia (ITP) patients.* Methods*. Peripheral blood mononuclear cells from ITP patients and controls were stimulated with PMA, ionomycin, and Brefeldin A. The frequencies of CD19^+^IL-10^+^ B cells, CD3^+^CD4^+^IL-17^+^ Th17 cells, and CD4^+^CD25^hi^Foxp3^+^ Treg cells were analyzed by flow cytometry. The mRNA expression of Foxp3 and ROR*γ*t was detected by real-time quantitative PCR.* Results*. The number of B10 cells was elevated in ITP patients. After first-line therapies, it remained at high level in patients who achieved complete or partial response but decreased in those who acquired no response. There was a positive correlation between B10 cells and Tregs in ITP both before and after therapies. The ratio of Treg/Th17 decreased in ITP, and it strongly correlated with B10 cells.* Conclusions*. The frequency of B10 cells is elevated in ITP and it correlates with both the Tregs counts and the Treg/Th17 ratio. B10 cells to regulate functional T cell subsets might be impaired in patients with ITP.

## 1. Introduction

Primary immune thrombocytopenia (ITP) is an acquired immune-mediated bleeding disorder characterized by isolated thrombocytopenia due to increased platelet clearance and/or impaired thrombocytogenesis [1–3]. Both abnormal B cell-dependent humoral immune responses and deviant differentiation of T cell subsets have been well described in patients with ITP, such as the existence of autoantibodies against platelet glycoproteins [4, 5], skewed Th1/Th2 ratio [6, 7], decreased number and impaired functions of regulatory T cells (Tregs) [8, 9], and increased frequency of Th17 cells [10, 11].

Generally speaking, pathogenic B cells are driven by platelet-specific helper T cells to proliferate into autoantibody-secreting cells and thus are thought to be responsible for decreased peripheral blood platelet count in patients with ITP [12]. Rituximab, an anti-CD20 monoclonal antibody that depletes B cells, has been used to treat ITP for about a decade and has shown its efficacy in a subpopulation of ITP patients [13, 14]. Interestingly, successful rituximab treatment not only depletes CD20^+^ B cells, but also affects Th1 and Treg subsets [15, 16], suggesting complicated interaction between B and T immune cells in ITP patients.

Recent studies have evidenced that specific B cell subsets, so-called regulatory B cells (Bregs), are potent immune response regulators and play important roles in autoimmune diseases [17–19]. A number of regulatory B cell subsets have been reported, among which the most well characterized is interleukin-10-producing B cell subset (B10 cells) [20, 21]. Heretofore, there is no specific transcription factor or unique phenotypic markers for B10 cells. The identification of B10 cells is now dependent on their ability to produce interleukin-10 (IL-10), an anti-inflammatory cytokine that contributes to the regulatory functions of B10 cells. Recent findings have shown that IL-10 produced by B10 cells is required in combination with different costimulatory molecules for the differentiation and maintenance of Treg and for inhibition of Th17 [22, 23]. Importantly, the Treg/Th17 ratio is relevant to the clinical diversity of ITP and it might predict long-term outcome in ITP patients [10]. It is reasonable to speculate that B10 cells are of importance for the Treg/Th17 balance; thus the numerical and/or functional deficiencies in B10 cells might be clinically relevant in patients with ITP.

Studies in a variety of mouse models of immune-mediated disorders have demonstrated that B10 cells are of crucial importance in preventing disease development and ameliorating established symptoms [24–27]. In human, it has also been shown that the frequencies of B10 cells are correlated with disease activity in rheumatoid arthritis (RA) [28] and chronic hepatitis B virus infection [29]. However, little is known about the characteristics of B10 cells in ITP. Given the important immune regulatory functions of B10 cells and the broken immune homeostasis in ITP, to better dissect the features of B10 cells is essential both for the understanding of the pathogenesis of ITP and for the development of new treatment strategies. In this study, we investigated the profile of circulating IL-10-producing B cell subset in newly diagnosed ITP patients both before and after first-line therapies, as well as its role in maintaining the balance of Treg/Th17.

## 2. Materials and Methods 

### 2.1. Patients and Controls

Thirty-five adult patients with newly diagnosed ITP were enrolled in this study between September 2012 and May 2013 from Zhongshan Hospital, Fudan University. All patients required treatment due to clinically significant bleeding and/or platelet count less than 20 × 10^9^/L. Secondary ITP, pregnant patients, and those who were complicated with contraindications to glucocorticoid therapy were excluded. All patients received first-line glucocorticosteroid (GC) treatment with or without intravenous immunoglobulin depending on the severity of bleeding symptoms. Blood samples were collected prior to and 4 weeks after initial administration of GCs when responses were evaluated. Responses to GCs were classified into complete response (CR), partial response (PR), and no response (NR), according to the response criteria of ITP proposed by an international working group [30]. The clinical characteristics of patients are listed in Table 1. The healthy control group consisted of 25 adult volunteers (17 females and 8 males, age range 21–74 years, median 47 years). Platelet counts ranged from 187 to 294 × 10^9^/L, with the median of 239 × 10^9^/L.

Ethical approval for this study was granted by Medical Ethics Committees of Zhongshan Hospital, Fudan University. Written informed consent was obtained from each patient in accordance with the Declaration of Helsinki.

### 2.2. Isolation of Peripheral Blood Mononuclear Cells (PBMCs)

PBMCs were isolated from 6 mL ethylenediaminetetraacetic acid-treated venous blood samples by Ficoll-Hypaque gradient centrifugation (2,200 rpm at room temperature for 15 min). Washed and resuspended, PBMCs were cryopreserved in fetal bovine serum containing 10% dimethyl sufloxide (DMSO) and stored in liquid nitrogen for future cell culture, flow cytometric analysis, and real-time polymerase chain reaction.

### 2.3. Cell Culture

Cryopreserved PBMCs were thawed at 37°C and washed twice with Hank's balanced salt solution (HBSS). PBMCs were seeded at 5 × 10^5^/mL in 24-well plates in RPMI1640 medium supplemented with 10% heat-inactivated fetal bovine serum, 2 mM L-glutamine, 200 U/mL penicillin, and 100 *μ*g/mL streptomycin. For intracellular staining of IL-10 and IL-17, cells were stimulated with 50 ng/mL phorbol-12-myristate-13-acetate (PMA) (Sigma-Aldrich, USA) and 500 ng/mL ionomycin (Sigma-Aldrich, USA) for 24 h (IL-10) or 6 h (IL-17) in the presence of 1 mM brefeldin A (BFA) (BioLegend, USA) for the last 4 h of culture.

### 2.4. Flow Cytometric Analysis

PBMCs were stained with PE-conjugated anti-CD25 and PE-Cy5-conjugated anti-CD4 or isotypes (BioLegend, USA) for 20 min at 4°C, washed twice, fixed, and permeabilized and then stained with Alexa Fluor 488-conjugated anti-Foxp3 for analysis of Treg subpopulation. For B10 and Th17 detection, cultured cells were stained with surface FITC-conjugated anti-CD19 and intracellular APC-conjugated anti-IL-10 (B10) or FITC-conjugated anti-CD3, PE-conjugated anti-CD4, and intracellular Alexa Fluor 647-conjugated anti-IL-17 (Th17). Acquisitions were performed on a FACS AricII flow cytometer (BD, USA) and were analyzed using Flowjo software version 7.6.1.

### 2.5. Real-Time Polymerase Chain Reaction (RT-PCR)

Total RNA was extracted from PBMCs with Trizol reagent (Invitrogen, USA) and converted into cDNA using a PrimeScript RT reagent kit (Takara, Japan) according to the manufacturer's instructions. The mRNA expression of Foxp3 and ROR*γ*t was quantified using the SYBR Premix Ex Taq (Takara, Japan) on a MasterCycler Realplex^4^ system (Eppendorf, German), with GAPDH expression as a control. Amplification was performed in a total volume of 20 *μ*L for 40 cycles of 5 s at 95°C and 30 s at 60°C after initial denaturation (95°C, 30 s). The primer sequences were as follows: GAPDH forward: 5′-GGTGGTCTCCTCTGACTTCAACA-3′; GAPDH reverse: 5′-GTTGCTGTAGCCAAATTCGTTGT-3′; Foxp3 forward: 5′-GTGGCATCATCCGACAAGG-3′; Foxp3 reverse: 5′-TGTGGAGGAACTCTGGGAAT-3′; ROR*γ*t forward: 5′-GTGCTGGTTAGGATGTGCCG-3′; ROR*γ*t reverse: 5′-GTGGGAGAAGTCAAAGATGGA-3′. Samples were analyzed in triplicate; 2^−ΔΔCt^ was used to calculate fold change of mRNA expression.

### 2.6. Statistical Analysis

All analyses were performed with STATA 10.0 software. Data were expressed as mean ± SD. Normality was assessed by Shapiro-Wilk test. Student's *t*-test and Wilcoxon rank-sum test were used for data which fulfilled normal distribution and for those which did not, respectively. Paired Student's *t*-test was used to evaluate the differences between patients before and after first-line therapies. When multiple groups were compared, one-way ANOVA and Kruskal Wallis test were used for data which fulfilled normal distribution and for those which did not, respectively. The correlation between B10 and T cell subsets was estimated by linear regression. For all tests, two-sided *P* values less than 0.05 were considered statistically significant.

## 3. Results

### 3.1. Increased Frequency of IL-10-Producing B Cells in Patients with ITP

**Figure 1:**
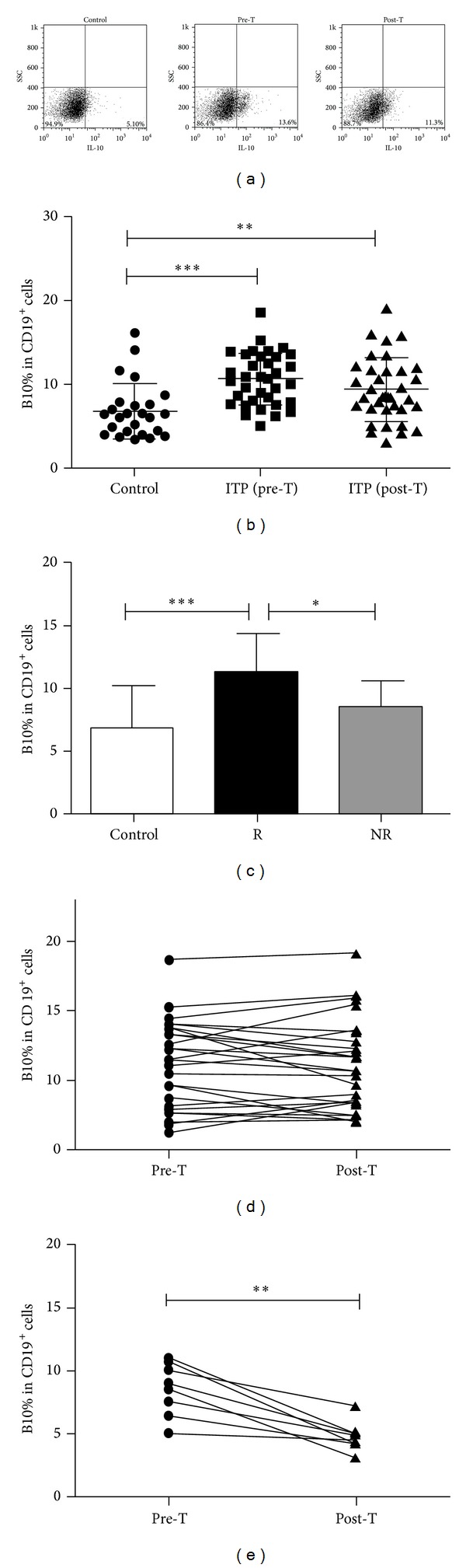
Elevated number of IL-10-producing B cells in patients with ITP. (a) Representative dot plots of IL-10-producing B cells (IL-10^+^, gated on CD19^+^ cells) in one healthy subject and one ITP patient before and after first-line therapies. (b) The percentage of IL-10-expressing B cells in total CD19^+^ B cells was higher than that in controls both before and after first-line therapies. (c) At the diagnosis of ITP, NR patients showed relative lower number of IL-10^+^CD19^+^ B cells when compared with R patients. (d) The percentage of B10 cells remained at a high level in R patients after first-line therapies. (e) The percentage of B10 cells decreased significantly in NR patients after first-line therapies. Pre-T: before treatment; post-T: after treatment. *P < 0.05; **P < 0.01; ***P < 0.001. NR: no response; R: response (including partial response and complete response).

When stimulated with PMA and ionomycin for 24 hours, PBMCs from ITP patients showed higher percentage of IL-10^+^CD19^+^ B cells in total CD19^+^ B lymphocytes than that from normal controls ((10.65 ± 3.11)% and (6.85 ± 3.31)%, resp.; *P* < 0.001) (Figures 1(a) and 1(b)). Similarly, the absolute number of IL-10^+^CD19^+^ B cells in ITP group was (1.58 ± 1.14) × 10^7^/L, which was also higher than that of (0.81 ± 0.45) × 10^7^/L in healthy subjects (*P* < 0.001).

There were eight out of the thirty-five patients who showed resistance to first-line therapies. We further compared the difference in B10 cells between NR and the rest of the patients. At the diagnosis of ITP, NR patients showed lower percentage of IL-10^+^CD19^+^ B cells when compared with patients who responded to the treatment ((8.52 ± 2.11)% and (11.29 ± 3.11)%, resp.; *P* = 0.025). While the percentage of IL-10^+^CD19^+^ B cells at diagnosis in CR/PR patients was significantly higher than that in controls (*P* < 0.001), it was comparable between NR patients and healthy subjects (*P* = 0.193) (Figure 1(c)).

### 3.2. The Number of IL-10-Producing B Cells Was Decreased after First-Line Therapies in NR Patients but Not in CR/PR Patients

After first-line therapies, both the frequency (9.53 ± 3.86)% and the absolute number (1.49 ± 1.29) × 10^7^/L of IL-10^+^CD19^+^ B cells remained at high levels, either of which was comparable to that before treatment (*P* = 0.183 and *P* = 0.788, resp.) and was significantly higher than that in normal controls (Figures 1(a) and 1(b)).

It was interesting that while the number of B10 cells was not altered after first-line therapies in CR/PR patients ((11.29 ± 3.11)% versus (10.93 ± 3.17)%; *P* = 0.310) (Figure 1(d)), it decreased in NR patients to a level of (4.77 ± 1.17)% (Figure 1(e)), which was even lower than that in controls, although the difference was not statistically significant (*P* = 0.099).

### 3.3. Positive Correlation between IL-10-Producing B Cells and Tregs in ITP Patients

**Figure 2:**
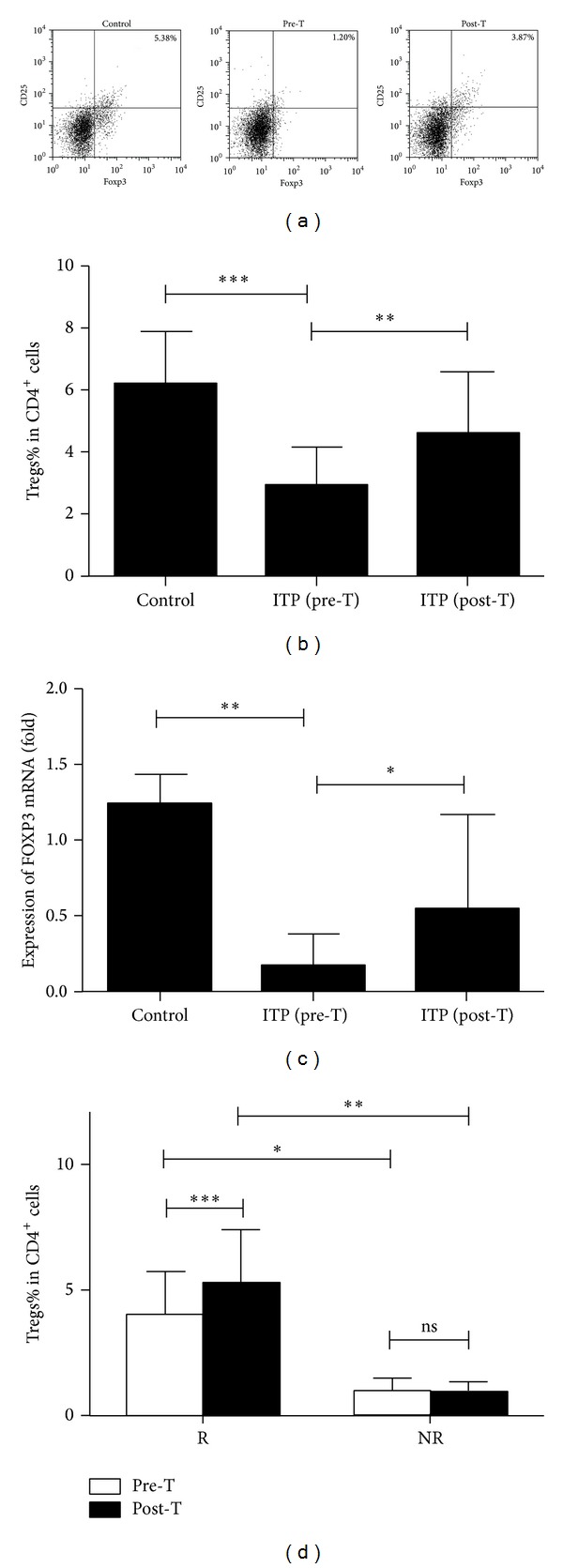
Decreased number of Tregs in patients with ITP. (a) Representative dot plots of Tregs (CD25^+^Foxp3^+^, gated on CD4^+^ cells) in one healthy subject and one ITP patient before and after first-line therapies. (b) Decreased frequency of Tregs can be improved by first-line therapies in ITP patients. (c) The expression of FOXP3 mRNA was also decreased in ITP patients. (d) The deficiency in the number of Tregs was corrected by first-line therapies in R patients, but not in NR patients. *P < 0.05; **P < 0.01; ***P < 0.001.

Some studies have suggested that B10 cells facilitate the differentiation and expansion of Tregs, which prompted us to look into the effects of increased IL-10^+^CD19^+^ B cells on Tregs in ITP patients. Quite contrary to increased number of IL-10^+^CD19^+^ B cells, the percentage of CD4^+^CD25^high^FOXP3^+^ Tregs was significantly decreased in newly-diagnosed ITP patients when compared with that in normal controls ((2.87 ± 1.22)% versus (6.17 ± 1.67)%; *P* < 0.001) (Figures 2(a) and 2(b)). Accordingly, the absolute number of Tregs was also diminished in ITP patients ((2.29 ± 0.99) versus (5.54 ± 1.48) × 10^7^/L; *P* < 0.001). The expression of FOXP3 mRNA was downregulated in ITP patients, which was in accordance with the results of flow cytometry analyses (Figure 2(c)).

**Figure 3:**
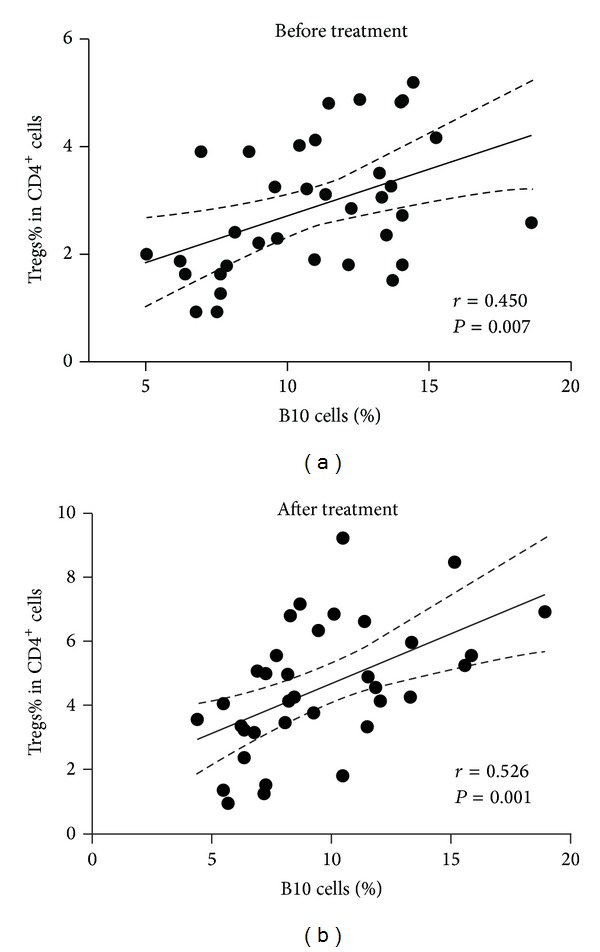
Positive correlations between B10 cells and Tregs in ITP patients before (a) and after (b) first-line therapies.

No correlation was found between the frequency of IL-10^+^CD19^+^ B cells and Tregs in the healthy controls (*r* = 0.141, *P* = 0.501). However, within newly diagnosed ITP group, both the percentage and the absolute number of B10 cells were positively correlated with those of Tregs (percentage: *r* = 0.450, *P* = 0.007; absolute number: *r* = 0.490, *P* = 0.005) (Figure 3(a)). After first-line therapies, the number of Tregs increased but was still lower than that in controls (Figures 2(a) and 2(b)). Similar to the results observed in B10 cells, the percentage of Tregs in NR and CR/PR patients changed differently in response to the first-line therapies: Treg% in posttreatment NR patients remained unchanged ((1.03 ± 0.43)% versus (0.92 ± 0.41)%; *P* > 0.05), while that in CR/PR ones increased significantly ((5.27 ± 1.09)% versus (4.08 ± 1.67)%; *P* < 0.001) (Figure 2(d)). The positive correlation between B10 cells and Tregs still existed in posttreatment patients (percentage: *r* = 0.526, *P* = 0.001; absolute number: *r* = 0.366, *P* = 0.030) (Figure 3(b)).

### 3.4. IL-10-Producing B Cells Correlated with Treg/Th17 Ratio in ITP Patients

**Figure 4:**
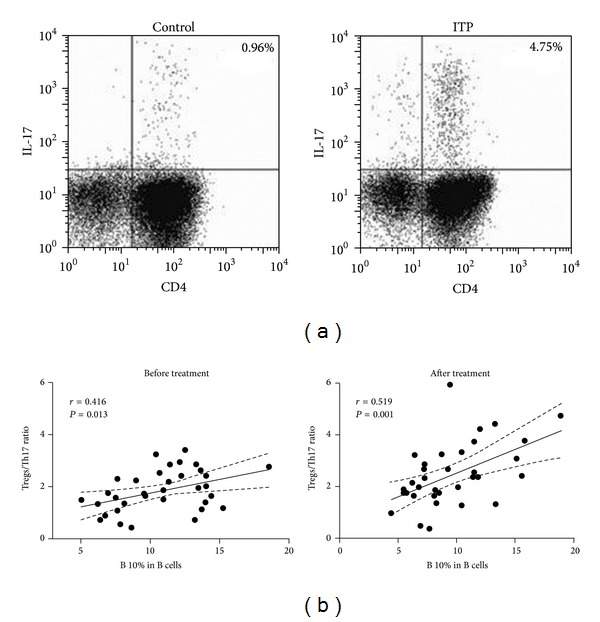
Correlation between B10 cells and Treg/Th17 ratio in ITP patients. (a) Representative dot plots of Th17 (CD4^+^IL-17^+^, gated on CD3^+^ cells) in one healthy subject and one ITP patient. (b) Positive correlations between IL-10-producing B cells and Treg/Th17 ratio in ITP patients before (left) and after (right) first-line therapies.

The frequency of CD3^+^CD4^+^IL-17^+^ (Th17) cells, a proinflammatory T cell subset, was also significantly elevated in ITP patients (NC, (0.83 ± 0.40)%; ITP, (2.46 ± 1.09)%, *P* < 0.001) (Figure 4(a)), which was reminiscent of the enriched IL-10^+^CD19^+^ B cells in ITP. The expression of ROR*γ*t mRNA was also upregulated in patients with ITP. Nevertheless, no correlation was found between the percentages of Th17 and B10 cells in ITP patients (*r* = 0.201, *P* = 0.231), nor between the absolute numbers of these two subsets (*r* = 0.245, *P* = 0.156). Meanwhile, there was no difference between CR/PR and NR patients concerning the percentage of Th17 cells (R, (2.44 ± 1.43)%; NR, (3.12 ± 1.57)%; *P* = 0.256). After first-line therapies, both CR/PR and NR patients showed as high Th17 cell numbers as before the treatment (R, (1.91 ± 1.03)% versus (2.44 ± 1.43)%, *P* = 0.129; NR, (3.18 ± 1.74)% versus (3.12 ± 1.57)%, *P* = 0.943). Still, the elevated number of Th17 cells in posttreatment patients was not correlated with IL-10-producing B cells (*r* = 0.114, *P* = 0.250).

As the Treg/Th17 imbalance plays an important role in the pathogenesis of ITP, we further analyzed the potential correlation between B10 cells and the ratio of Treg/Th17. As expected, Treg/Th17 ratio was significantly decreased in untreated ITP patients. There was no correlation between the number of B10 cells and Treg/Th17 ratio in normal controls. However, a positive correlation was found between the two parameters in ITP patients (*r* = 0.416, *P* = 0.013). The positive correlation was even stronger after first-line therapies (*r* = 0.519, *P* = 0.001) (Figure 4(b)).

## 4. Discussion

Human immune system is a sophisticated one in which pro- and anti-immune cells/cytokines influence and constrain each other to keep a delicate balance. The functional and numerical deficiencies in regulatory immune cells are to a great extent responsible for the disruption of immune homeostasis in autoimmune disorders, such as ITP. The present study shows that, in addition to regulatory T cells, the number of circulating IL-10-producing B cells is also eccentric in ITP patients, indicating that both regulatory B and T cells participate in the development and perpetuation of ITP.

Our data show that the number of circulating B10 cells is significantly increased in ITP patients at the diagnosis of the disease, which is in line with results from some other studies on autoimmune disorders such as systemic lupus erythematosus, rheumatoid arthritis, and Sjogren's syndrome [31–33]. However, when clinical response to first-line therapies was taken into consideration, we found that the number of B10 cells was relatively lower in NR patients at the diagnosis of ITP. In addition, while the number of IL-10-producing B cells was not altered in CR or PR patients after first-line therapies, it dropped to a level in NR patients even lower than that in controls. Recently, Li et al. also reported reduced levels of IL-10 expression in CD19^+^ B cells in a cohort of refractory ITP patients [34]. These data suggest that the number of IL-10-producing B cells might correlate with clinical outcome in ITP patients.

Multiple studies have demonstrated that B10 cells are able to upregulate the expression of Foxp3 and thus expand the population of Tregs [22, 23, 35]. However, no correlation was found between B10 cells and Tregs in healthy controls. Wilde et al. also evaluated the correlation between these two cell subsets; they found that although B10 cells positively correlated with Tregs in untreated ANCA-associated vasculitis patients with quiescent disease, no association was found in healthy controls [36]. In the current study, we confirmed that CD4^+^CD25^+^Foxp3^+^ Treg subset is numerically deficient in ITP. However, there is a positive correlation between the frequency of IL-10-producing B cells and CD4^+^CD25^+^Foxp3^+^ Tregs in newly diagnosed ITP patients. The opposite changes in B10 cells and Tregs, together with the positive correlation between the two subsets, imply that the ability of B10 cells to enlarge the population of Tregs might be insufficient in newly diagnosed ITP patients. After first-line therapies, the number of B10 cells seemed to be stable, while Tregs increased significantly. Although these two cell subsets responded to first-line therapies differently, the positive correlation still existed in the group of posttreatment ITP patients, which might imply that the regulatory function of B10 cells on Tregs was restored, at least partially, by first-line therapies.

We also observed that the frequency of CD3^+^CD4^+^IL-17^+^ Th17 cells was elevated in patients with ITP. However, the number of Th17 cells seemed somehow to be fixed during the course of ITP as neither did it fluctuate after first-line therapies, nor did it differ between NR and R patients. Some studies suggest that human IL-10-producing B cells do not affect Th17 differentiation [32, 35]. In contrast, Flores-Borja et al. have recently demonstrated that healthy CD19^+^CD24^hi^CD38^hi^ B cells, a subset of B cells that produce a large amount of IL-10, do inhibit naïve T cell differentiation into Th17 cells [37]. In the present study, we cannot find any correlation between IL-10^+^CD19^+^ B cells and Th17 cells, indicating that the ability of B10 cells to inhibit Th17, if any, is largely dampened in ITP. Interestingly enough, there is a strong correlation between B10 cells and Treg/Th17 ratio in ITP patients. The Treg/Th17 ratio is believed to be relevant to the clinical diversity of ITP and it might have prognostic role in ITP patients [10], which is in accordance with the result that the number of B10 cells is relatively lower in NR patients than that in CR/PR patients. That is, despite the impaired functions of B10 subset in orchestrating Treg and Th17 cells in ITP, it might provide useful information for clinical management and therapeutic options.

Taken together, our data demonstrated the increased frequency of IL-10^+^CD19^+^ B cells and its association with essential T cell subsets in patients with ITP. Considering immune regulatory functions of B10 cells, a conflict exists between the increased number of IL-10^+^CD19^+^ B cells and the disrupted immune homeostasis in ITP. Lemoine et al. proposed that the activation signals from T cells initiate regulatory properties in B cells that modulate T cell responses [33], forming a negative feedback loop involving T cell activation and regulatory B cells. In this feedback model, immune hyperreaction raises signals, directly or indirectly, to drive IL-10-producing B cells to expand, which in turn suppresses immune responses. In physiological conditions, the feedback loop works well and keeps a subtle balance in immune system, while, in ITP, IL-10-producing B cells might be no longer competent for immune regulation; consequently the feedback loop is disrupted and immune overreaction may occur. We have previously demonstrated that the secretion of IL-10 by a subset of B cells is impaired in ITP patients [38], which might lead to functional impairment of B10 cells. However, the underlying mechanism of impaired IL-10 secretion in ITP is currently unknown. Further studies that exactly dissect the properties of regulatory B cells in ITP are obviously warranted.

## 5. Conclusion

In addition to Treg and Th17 cells, the frequency of IL-10-producing B cells is also aberrant in patients with ITP. NR patients show relative lower number of B10 cells both before and after first-line therapies, indicating that B10 cells might associate with clinical outcome in ITP patients. B10 cells positively correlate with both Treg cells and Treg/Th17 ratio. However, the elevated number of B10 cells together with Treg/Th17 imbalance suggests that the ability of B10 cells to regulate functional T cell subsets might be insufficient in patients with ITP. The role of B10 cells for the development and perpetuation of ITP needs to be further investigated.

## Figures and Tables

**Table 1:** Clinical characteristics of ITP patients.

Patient number	Sex	Age (years)	Duration of disease (months)*	Platelet counts (×10^9^/L)		Response to first-line therapies
				Before treatment	After treatment	
1	F	56	6	8	6	NR
2	F	28	8	11	56	PR
3	M	38	2	12	26	NR
4	F	63	1	18	84	PR
5	F	47	12	9	45	PR
6	F	67	5	2	169	CR
7	M	46	20	8	227	CR
8	F	52	1	13	175	CR
9	M	19	18	7	239	CR
10	F	34	14	3	18	NR
11	M	41	7	17	157	CR
12	F	68	21	6	193	CR
13	F	43	2	5	296	CR
14	F	36	4	12	137	CR
15	F	50	14	14	15	NR
16	F	79	4	4	46	PR
17	M	35	1	1	6	NR
18	F	38	5	8	186	CR
19	F	59	9	15	24	NR
20	F	67	32	22	47	PR
21	M	33	1	3	134	CR
22	F	73	15	18	214	CR
23	M	24	1	7	151	CR
24	F	24	1	6	86	PR
25	F	50	4	8	65	PR
26	M	50	20	12	26	NR
27	F	30	12	8	38	PR
28	F	28	8	16	64	PR
29	M	46	32	20	84	PR
30	F	54	16	13	23	NR
31	M	62	2	7	141	CR
32	F	49	6	11	137	CR
33	F	35	10	3	192	CR
34	F	37	1	12	179	CR
35	F	51	4	17	231	CR
Median	—	46	6	9	137	—
(Min.–max.)	—	(19–79)	(1–32)	(1–22)	(6–296)	—

*Approximately estimated according to initial manifestation of bleeding symptoms and/or platelet count <100 (×10^9^/L).

## Abbreviations

ITP: Immune thrombocytopenia
IL-10: Interleukin-10
Breg: Regulatory B cell
Treg: Regulatory T cell
GC: Glucocorticosteroid
PBMCs: Peripheral blood mononuclear cells
RT-PCR: Real-time quantitative polymerase chain reaction.
